# Mechanical and Antimicrobial Properties of the Graphene-Polyamide 6 Composite

**DOI:** 10.3390/ma17143465

**Published:** 2024-07-12

**Authors:** Paweł Głuchowski, Marta Macieja, Robert Tomala, Mariusz Stefanski, Wiesław Stręk, Maciej Ptak, Damian Szymański, Konrad Szustakiewicz, Adam Junka, Bartłomiej Dudek

**Affiliations:** 1 Institute of Low Temperature and Structure Research Polish Academy of Sciences, PL-50422 Wroclaw, Poland; 246316@student.pwr.edu.pl (M.M.); r.tomala@intibs.pl (R.T.); m.stefanski@intibs.pl (M.S.); w.strek@intibs.pl (W.S.); m.ptak@intibs.pl (M.P.); d.szymanski@intibs.pl (D.S.); 2 Faculty of Chemistry, Wroclaw University of Science and Technology, PL-50370 Wroclaw, Poland; konrad.szustakiewicz@pwr.edu.pl; 3 Platform for Unique Model Application, Department of Pharmaceutical Microbiology and Parasitology Wroclaw Medical University, PL-50367 Wroclaw, Poland; feliks.junka@gmail.com (A.J.); bartlomiej.dudek@umw.edu.pl (B.D.)

**Keywords:** graphene flakes, PA6, polyamide, composite, mechanical properties, antimicrobial activity

## Abstract

This paper presents the synthesis and characterization of graphene–polymer composites, focusing on their mechanical and antibacterial properties. Graphene flakes were obtained via an electrochemical method and integrated into polyamide 6 (PA6) matrices using melt intercalation. Various characterization techniques confirmed the quality of the graphene flakes, including X-ray diffraction (XRD), Raman spectroscopy, and infrared (IR) spectroscopy, as well as scanning and transmission electron microscopy (SEM and TEM) imaging. Mechanical tests showed an increase in the elastic modulus with graphene incorporation, while the impact strength decreased. The SEM analysis highlighted the dispersion of the graphene flakes within the composites and their impact on fracture behavior. Antimicrobial tests demonstrated significant antibacterial properties of the composites, attributed to both oxidative stress and mechanical damage induced by the graphene flakes. The results suggest promising applications for graphene–polymer composites in advanced antimicrobial materials.

## 1. Introduction

Graphene, a single layer of graphite, takes the form of a two-dimensional structure in which sp^2^ hybridization occurs between carbon atoms [1]. This unique structure contributes to exceptional properties, including excellent mechanical, thermal, and electrical characteristics. Graphene, as an allotropic form of carbon, boasts a mechanical strength approximately 100 times greater than steel. Its high electron mobility and resistance contribute to outstanding conductivity. Due to these distinctive properties, graphene has captured the interest of numerous scientists. While there are various laboratory methods for synthesizing graphene, producing it on an industrial scale remains a challenge. The preparation method of graphene plays a crucial role in determining its properties and, consequently, its potential applications. Among the diverse techniques available for graphene production, the focus on graphene flake preparation has gained prominence due to its relevance in achieving scalable and versatile graphene structures. The selection of an appropriate preparation technique significantly influences the size, morphology, and quality of the graphene flakes, thereby influencing their performance in specific applications. Among others, the main production techniques involve mechanical exfoliation [2], chemical exfoliation (Hummers’ Method) [3], chemical vapor deposition (CVD) [4], liquid phase exfoliation [5], hydrothermal and solvothermal synthesis [6], and electrochemical exfoliation [7]. The choice of a specific preparation method depends on the intended application, scalability requirements, and desired graphene properties. Researchers continue to explore and develop new techniques to enhance the efficiency, scalability, and quality of graphene flakes for diverse applications in electronics, energy storage, sensors, and composite materials [8,9,10,11].

In recent years, there has been a consistent growth in interest regarding composites that incorporate various types of carbon. Fillers such as carbon nanotubes (CNTs), fullerenes, black carbon, graphite, graphene, and graphene oxide (GO) are employed [12,13,14,15]. The use of nanosized particles as fillers allows the composites to retain favorable processing properties and a low weight, while also acquiring new, intriguing, and often unforeseen properties. Wu et al. [16] showed that a composite of CNT with a PVA reinforced the PVA matrix and improved the strength from 50 to 1255 MPa and the electrical conductivity from 0 to 1948 S cm^−1^. Miao et al. [17] revealed in their review the positive impact of CNT addition on the wear resistance of polymers. Kausar [18] demonstrated that composites based on fullerene and a polymer matrix may be utilized in energy storage applications, and that among nanocarbon nanofillers, fullerene has big potential to develop high-performance conductive polymeric matrices [19]. The addition of cheap black carbon to the polymers usually leads to an improvement in the electrical conductivity of the composites [20,21]. The graphite-based composites show improvement in their tribological [22], as well as biological [23], properties. But among all the other carbon structures, graphene and graphene oxide attract the most attention. The incorporation of graphene into composites offers several advantages, leading to improvements in strength, conductivity, and other physical, chemical, and biological properties [24]. The addition of graphene to polymer matrices enhances the mechanical strength of the resulting composites, such as tensile strength and stiffness. For the poly (vinyl alcohol)/graphene composite, the tensile strength increased by 150% compared with the pure polymer. The content of graphene in the composite was 1.8 vol.% graphene loading [25]. Graphene flakes in polymers may also improve the heat dissipation capabilities of composites, as well as the electrical conductivity, which can be advantageous in electronic applications and energy storage. The low density of graphene contributes to the development of lightweight composites, making them suitable for aerospace and automotive applications. Additionally, the addition of graphene to matrix composites enhances corrosion resistance and wettability, making the composites more durable for work in aggressive environments. Another important aspect is the application of graphene flake-based composites in biomedical applications [26], where they are used due to their biocompatibility and low cytotoxicity. Antibacterial and antibiofilm properties are promising features of graphene and its derivatives. Due to the molecule structure of graphene oxide (GO), this graphene derivative causes bacterial cell degradation [27]. The versatility of graphene in enhancing the various properties of composite materials has led to extensive research and development in this field. Many reports considering the antibacterial properties of polymeric materials based on graphene and its derivatives can be found in the literature. Authors have proposed the use of many different polymers to obtain composites. It turns out that depending on the fabricated composite, it is possible to obtain different values of the killing rate at very different times depending on the requirements of potential applications. Taking the pathogen *E. coli* as an example, it was found that the use of a PVA (poly(vinyl alcohol)) [28] or PLA (poly(lactic acid)) [29] polymer leads to the termination of more than 99% of bacteria within 24 h. Investigations show that the PLLA (poly(l-lactic acid)) polymer is effective in reducing the bacterial termination time to 12 h with a kill rate of 90% [30]. On the other hand, outstanding results are presented by the PSPH (poly[5,5-dimethyl-3-(3′-triethoxysilylpropyl)hydantoin])/GO composite, which reduces the number of bacteria by 97% even in 30 min [31]. Researchers report that the mechanism of antibacterial activity may be based, among others, on the extraction of phospholipids from the bacterial membrane [32], oxidative stress [33], the insertion mode of action [34,35], or the production of reactive oxygen species [36]. Graphene and its derivatives also have great fungicidal properties that are related to their sharp edges, which result in stressing the cell membrane of the pathogen [36]. The laser-induced GO surface was also the subject of the investigation, which led to the photothermal treatment of wounds to protect them from fungi [37].

Polyamide is a synthetic material that is widely used in industry. It is known for its universal features and relatively low price. Several types of polyamide can be distinguished, which differ in their properties. Among them, PA6 is a very popular material, which is characterized by its excellent mechanical properties, and offers good chemical resistance, is highly thermoplastic, and is often used in the production of composites to improve its already good features or provide completely new ones [38,39,40]. The introduction of graphene into polyamide results in increased thermal and electrical conductivity, flame retardance, weight loss, and mechanical strengthening [39,41,42]. Moreover, the addition of graphene to polymers can lead to giving such composites antimicrobial properties [43]. The reinforced composites prepared in this way can be successfully used in aerospace, automobile, special types of engineering plastic, and 3D printing [44,45].

This paper presents the synthesis, mechanical, and biological characterization of graphene–PA6 composites, focusing on the influence of the graphene flake concentration on their properties. This study explores the antibacterial properties of composites incorporating graphene and examines how graphene additions affect their mechanical characteristics. Unlike previous research on biocomposites with graphene, our materials are composed exclusively of graphene flakes, without other bactericidal agents such as TiO_2_, ZnO, or Ag. We have developed an innovative method for producing these graphene flakes in large quantities, facilitating the scalable production of such composites. The use of the PA6 polymer in the composite matrix makes these materials suitable for 3D printing applications. The biocidal properties have been specifically tested against *Staphylococcus aureus* bacteria, indicating the potential for future use in manufacturing components for medical equipment. The results provide valuable insights into the multifaceted properties of graphene–polymer composites and their potential in various applications.

## 2. Experimental

### 2.1. Materials

The graphene flakes were obtained using the electrochemical method [7]. A strip of graphite foil (GFC99M1, Sinograf Toruń S.A., Toruń, Poland) serving as a cathode was placed in a beaker containing an aqueous solution of 0.1 M ammonium sulfate ((NH_4_)_2_SO_4_, Sigma Aldrich, as a part of chemical conglomerate Merck Group, Burlington, MA, USA), ≥99.0% with a single graphite block (serving as an anode) positioned between them. The anode activation was performed for 10 min at 2.5 V. Following this, the voltage was increased to 10 V and the current to 2.5 A, continuing the electrolysis for 1 h. Upon completion, the resulting solution containing the graphene flakes was filtered through a soft filter and washed five times with distilled water. The electrolysis was carried out in a salt solution under a constant voltage and current. An ultrasonic head (Tefic Biotech Co., Limited, Xi’an, China, Laboratory Ultrasonoficator TF-900N) was used to disperse the solutions with graphene. Water was removed from the graphene samples using a freeze-drying process (Tefic Biotech Co., Limited, Xi’an, China Freeze-dryer TF-10A). The prepared flakes were additionally dried in a vacuum dryer (CHEMLAND 06-DZ-2 BC, Starogard, Poland) before introducing them into the polymer to avoid water contamination.

The graphene flakes were introduced into the polyamide 6 matrix (polyamide 6 with a melt flow index of 120 cm^3^/10 min (5 kg; 270 °C) and a density of d = 1.14 g cm^3^ under the trade name Tarnamid T-27, supplied by Grupa Azoty S.A., Pułąwy, Poland), Tarnamid T-30, Grupa Azoty S.A., Pułąwy, Poland) using the melt mixing method. Polymer composites were produced with a graphene content equal to 0.1%, 0.5%, and 1% (weight percentage). All the materials were dried prior to extrusion under a vacuum (24 h, 60 °C). The dried material was divided into 10 g samples. Each sample contained PA6 pellets and graphene powder of the target composition. Then, the samples were mixed with a spatula and dosed by hand onto the first zone of the extruder.

Extrusion and granulation were performed using a Thermo Fisher Scientific Process 11 twin-screw extruder (Thermo Scientific, Waltham, MA, USA) with a screw diameter of 11 mm and L/d = 40. The temperature of the extruder barrel during the extrusion process was 240 °C and the screw speed was set at 200 rpm. There were nine zones in our extruder. The first was the dispensing zone and was unheated, zones 2–8 were heated, and the last zone, the head, was also heated. The machine was set up as follows: 260–255–250–245–245–240–240–230 (from hopper to die) and the stock temperature was 240 °C. After extrusion, the extrudate was cooled using an air track made of four fans (of our own manufacture) and granulated. The process yield was ~0.3 kg/h. All the materials were dried prior to extrusion under a vacuum (24 h, 60 °C).

**Figure 1:**
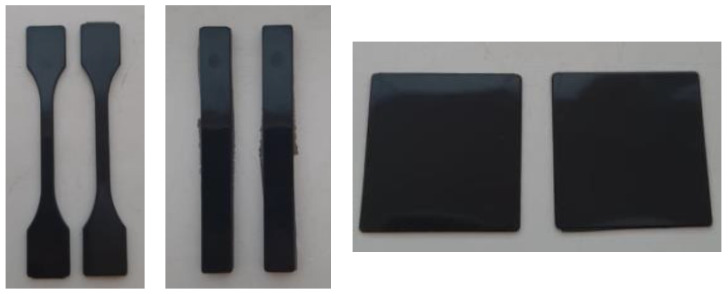
Standard shape 5A (**left side**) for the elasticity and tensile tests, standard shape 6A (**center**) for the impact tests, plates from which the discs were cut for the microbiological tests (**right side**).

The composite was used to prepare samples with the dimensions required by the PN-EN ISO 527-1 (PN-EN ISO 527-1, Plastics—Determination of tensile properties, Zurich, Switzerland, 2019) and PN-EN ISO 527-4 (PN-EN ISO 527-4, Plastics—Determination of tensile properties—Part 4: Test conditions for isotropic and orthotropic fibre-reinforced plastic composites, Zurich, Switzerland, 2019) procedures. A BOY XS screw injection molding machine (Dr. Boy GmbH & Co. KG, Neustadt-Fernthal, Germany) was used to produce the molded parts. The machine has three heating zones on the cylinder and heated channels. In this process, we set 250 °C in all zones and 60 °C was the mold temperature. The specimens for the tensile tests were injected using a 200 bar holding pressure (200 bar, 3 s), cooling time 15 s; the specimens for the Charpy impact tests were injected using a 200 bar holding pressure (200 bar, 5 s), cooling time 30 s; and the plates for the biological tests were injected using a 250 bars holding pressure (250 bar, 3 s), cooling time 10 s (250 °C, 200 bar). Normative shapes (5A from PN-EN ISO 527-4 for the tensile tests and for the Charpy impact tests according to PN-EN ISO 179-1) were prepared for the appropriate tests (Figure 1). For each mechanical test, eight samples were used.

The antimicrobial activity of the composites was tested in a 6-well plate (Biofil, Indore, India) using a microbiological spatula under aseptic conditions in a laminar chamber. The reference strains of methicillin-resistant *Staphylococcus aureus* 33591 (American Type and Culture Collection, Manassas, VA, USA) were incubated for 24 h at 37 °C in Tryptic Soy Broth (TSB, Biomaxima, Lublin, Poland). A suspension of the test strains was prepared in saline with a density of 0.5 McFarland (1.5 × 10^8^ CFU/mL) and diluted a thousand times in the culture medium. Next, 1 mL of the suspension was added to the wells of the 6-well plate with a composite disc located on the bottom of well and incubated for 24 h at 37 °C under static conditions. To control for microbial growth, the suspension was also added to the wells devoid of test substances in six replicates. After incubation, the medium was gently removed from the wells, and 1 mL of a 0.1% (*w*/*v*) solution of 3,2,5-triphenyltetrazolium chloride (TTC, Fluka, Buchs, Switzerland) in TSB was added to the wells. The system was then incubated again at 21 °C for 4 h. Next, the medium was removed from the plate wells, and 1 mL of a 9:1 mixture of methanol and acetic acid was added. The plate was incubated at room temperature for 30 min with shaking (350 RPM). From each well, 100 µL of the solution was withdrawn three times and transferred to three wells of a 96-well plate. The absorbance of the solutions was measured spectrophotometrically at a wavelength of 490 nm, and the absorbance of each sample was determined by calculating the average of three measurements.

### 2.2. Filler and Composite Characterization Methods

The structure of the flakes was characterized by X-ray diffraction measurements using Cu Kα monochromatic radiation (λ = 1.5406 Å) on an X’Pert PRO, PANalytical (Bruker, Karlsruhe, Germany), by scanning the diffraction angles (2θ) between 10° and 40°. The Raman spectrum was collected using a Renishaw InVia Raman spectrometer (Renishaw, Wotton-under-Edge, UK), a DM 2500 Leica microscope and 20× objective, a thermoelectrically cooled CCD, and an Ar^+^ laser operating at 514 nm as an excitation source. The IR spectrum in the mid-IR range of 4000–400 cm^−1^ was measured using a Nicolet iS50 infrared spectrometer (Thermo Fisher Scientific, Waltham, MA, USA) on the graphene flakes dispersed in the potassium bromide (KBr) pellet. Scanning (SEM) and transmission (TEM) electron microscopies, i.e., FEI NovaNano SEM 230 (FEI Company is part of Thermo Fisher Scientific, Waltham, MA, USA) and Philips CM-20 SuperTwin, Eindhoven, The Netherlands), were used to study the morphology of the graphene flakes and composites, respectively.

The tensile strength measurements were performed using an Instron 5966 static testing machine based on the PN-EN ISO 527 standard. The specimens were dried in an air dryer at 80 °C for 48 h. The stretching was performed at a constant speed (with 1 mm/min for the elastic modulus and 100 mm/min for the tensile strength and elongation for 5A specimens) along the main longitudinal axis. Impact tests were accomplished with a Zwick/Roell HIT5.5P impact hammer using a notched Charpy standard (PN EN ISO 527-1). The samples with notches cut along the long edge were used for the impact test. A 2J hammer was used. The edge of the Charpy hammer was struck once in the center of the sample.

The antibacterial properties of the composites were tested on the disks cut from the plates. For this purpose, 0.1% tetrazolium chloride (PanReac AppliChem, Barcelona, Spain) was introduced, which changes to red formazan as a result of the metabolic processes of live microorganisms. *Staphylococcus aureus* (No. 6538, reference collection from American Tissue and Cell Culture purchased from ATCC, Saint Cloud, MN, USA) was applied as a model microorganism. The red formazan crystals were dissolved using methanol (Chempur, Piekary Śląskie, Poland) and mechanical shaking at 450 rpm for 30 min (Schuttler MTS—4 shaker, IKA, Warszawa, Poland). Subsequently, the absorbance of the extracted formazan was measured spectrophotometrically (MultiScan Go spectrometer, Thermo Fisher Scientific, Waltham, MA, USA). An additional control test was a bacterial suspension exposed to the presence of 0.1% octenidine dihydrochloride (Schulke—Mayr, Norderstedt, Germany), the antiseptic agent with a strong antibacterial effect.

## 3. Results and Discussion

### 3.1. Structure and Morphology of the Graphene Flakes

**Figure 2:**
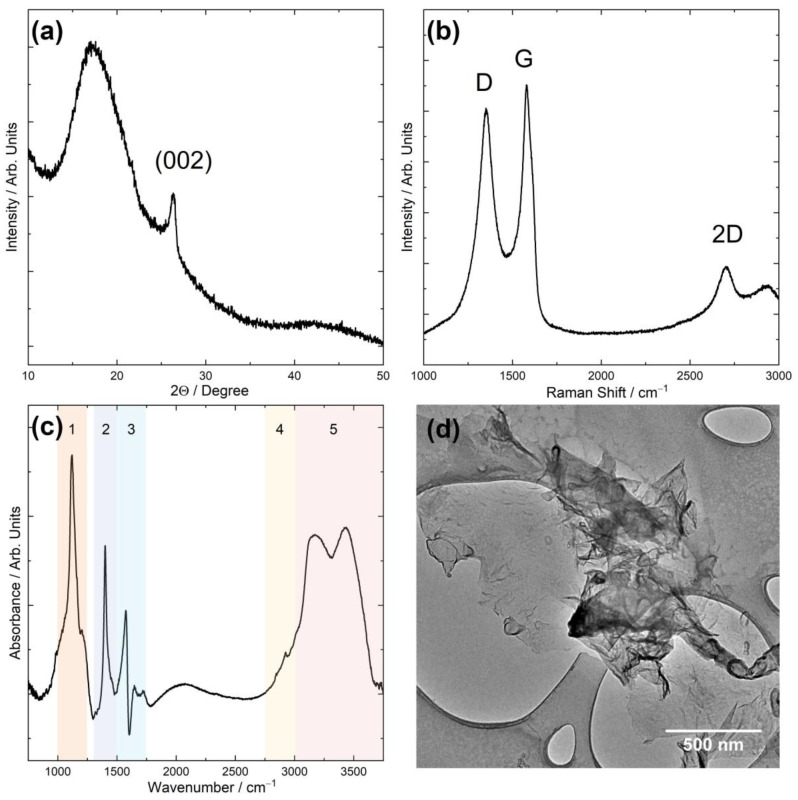
(**a**) XRD patterns, (**b**) Raman spectrum, (**c**) IR spectrum (the numbered areas correspond to the vibrations of individual bonds of functional groups on the graphene surface), and (**d**) TEM image of the graphene flakes used for composites.

The graphene flakes selected for the composite preparation underwent various characterization techniques to demonstrate their quality. In the XRD pattern (Figure 2a) of the analyzed samples, a peak is evident at approximately 2Ѳ = 26.5° (002 plane). Notably, this peak exhibits a considerable width in contrast to the characteristic spectrum of graphite, where this peak is sharp and exhibits a high intensity. This broadened peak is attributed to the interlayer spacing (0.34 nm) and can be assigned to the graphene nanoflakes [46]. The wide band observed in the 2Ѳ degree range from 10° to 22.5° suggests that part of the graphene flakes are present in the form of reduced graphene oxide (rGO) interspersed among the graphene layers. This observation implies the existence of oxygen groups localized at the layer edges, contributing to an increase in the spacing between the graphene layers. The Raman spectrum (Figure 2b) also confirms that the material obtained through electrochemical exfoliation, utilized as a filler for composites, exists in the form of graphene flakes. The spectrum exhibits repetitive patterns, revealing three distinct bands: the D band, located at about 1355 cm^−1^; the G band at 1585 cm^−1^; and the 2D band, observed in the range of 2702 cm^−1^ to 2709 cm^−1^. Notably, the heightened intensity of the 2D band and the emergence of the D band in the graphene spectrum strongly suggest the existence of surface defects in the material. For the graphene flakes, the degree of deformation was calculated as the ratio of the D-band to the G-band intensity, with this parameter valued at 0.13. The presence of an unsplit peak line further indicates that the graphene consists of a single layer, but the FWHM of the 2D band was 68.92 cm^−1^, suggesting that the graphene flakes have a structure consisting of approximately five layers. To measure the infrared spectrum (Figure 2c), the graphene flakes were ground with KBr and subsequently formed into a pellet. The signal of the tested sample appears to be relatively weak, possibly due to excessive light dispersion and absorption caused by the suspended graphene flakes in the pellet, and due to the hygroscopic nature of KBr. The additional bands above 3000 cm^−1^ (area 5) are present, attributed to the OH groups. The stretching vibrations of C-O and C=C are observed at 1130 cm^−1^ and 1577 cm^−1^, respectively. The band at ~1100 cm^−1^ (area 1) corresponds to the stretching C-O bonds, while the band at ~1375 cm^−1^ (area 2) is associated with the presence of the O-H deformation bond. Moreover, the bands within the wavenumber range from 1500 cm^−1^ to 1700 cm^−1^ (area 3) are presumed to arise from the water within the internal structure of the specimen. The bands between 2900 cm^−1^ and 3000 cm^−1^ (area 4) are likely related to the presence of organic impurities composed of aliphatic chains. The absorption measurement using infrared spectroscopy has not yielded a clear and unequivocal result. The use of non-dried potassium bromide and the inclusion of water content in the sample have made the results challenging to interpret. The TEM images of the graphene reveal that the lateral size of the flakes falls within the single-micrometer range from 1.6 to 2 µm, with some areas exhibiting a multilayer structure in the range of a few hundred nanometers (Figure 2d). The EDS analysis [7] shows that the C/O ratio was 7.7.

**Figure 3:**
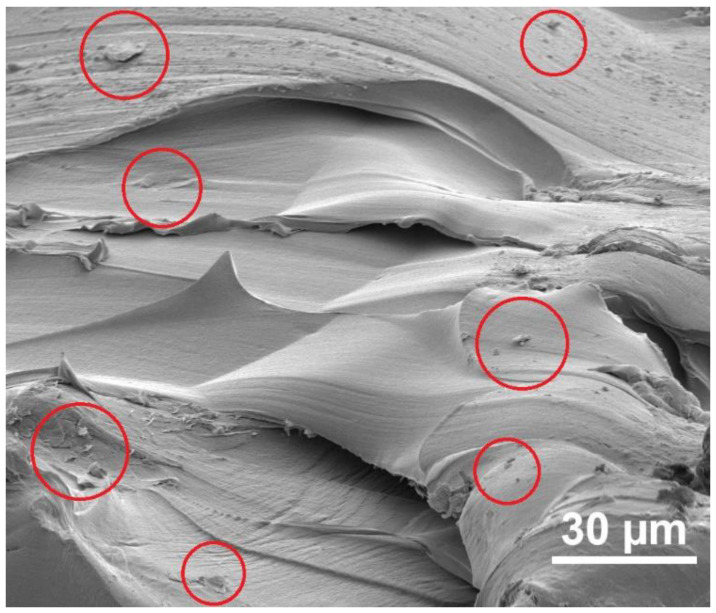
SEM image of the PA6 + 1 wt-% graphene composite. The red circles indicate the locations where the graphene flakes are present.

To illustrate the dispersion of the graphene flakes within the composite, scanning electron microscope (SEM) images were captured of a fractured section of a PA6 + 1 wt-% graphene composite (Figure 3). Despite the flakes possessing lateral dimensions below 1 µm, they are discernible only in select areas (red circles in Figure 3). Remarkably, even with a 1 wt-% inclusion of the flakes, there is no evident tendency for aggregation. In instances where the quantity of the flakes is smaller, the dispersion is further enhanced. However, due to their random dispersion and the low content of the flakes, this has a significant influence on the mechanical properties of the composites.

### 3.2. Mechanical Properties

**Figure 4:**
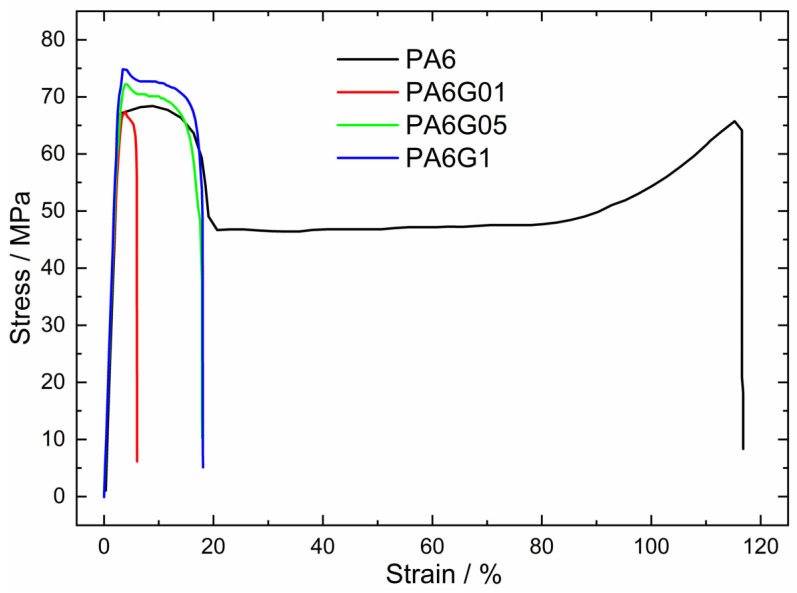
Stress–strain plot for PA6/graphene composites.

Tests were conducted on all composites to assess the influence of graphene addition on their mechanical properties. Based on the obtained results, an increase in the average longitudinal elastic modulus was observed with a rise in the concentration of graphene in the composites (Figure 4). The only exception was the composite with 0.1% graphene, which achieved an identical-to-pure-polymer average Young’s modulus value of 2560 MPa. In most cases, an increase in the amount of graphene filler led to an enhancement in this parameter, contributing to a greater resistance of the samples to static loads. Notably, the composite containing 1% graphene by weight exhibited the highest increase in the elastic modulus compared with pure PA6, reaching a significant improvement of 18% (Table 1). The maximum tensile stress transmitted by the structure increases in composites with reinforcement, except for samples with a graphene content of 0.1%, where the tensile strength is lower (66.7 MPa) than that of the pure polymer (69.4 MPa). Nanocomposites with 1% graphene by mass exhibit the highest tensile resistance, capable of transferring an average stress of 77.5 MPa. Interestingly, the properties of the composite based on the graphene flakes exhibit better parameters than those of the composite based on the carbon nanotubes. For example, the PA6/CNT composite (doped with 0.25% CNT) achieved a tensile strength of 74.3 MPa and a modulus of elasticity of 2440 MPa [47], whereas our composite (with 1% graphene flakes) achieved 77.5 MPa and 3020 MPa, respectively.

The measured impact strength for the reinforced samples, irrespective of the graphene content by weight, is observed to be lower than the impact strength of pure polyamide 6 (Table 2). The most significant decrease in impact strength is noted in the PA6/1% graphene nanocomposite, with a reduction of approximately 54%. Conversely, the PA6/0.5% graphene sample exhibits the smallest difference in impact strength compared with the reference samples, with a reduction of nearly 36%. A discernible trend is observed where the impact strength decreases with the decreasing graphene content, as evidenced by the lower impact strength for the 0.1% graphene content compared with 0.5%. The diminished impact strength in samples with lower graphene proportions in the composite may be attributed to imperceptible defects within the internal structure of certain shapes, leading to an increased material brittleness and consequential losses in bar integrity. The higher impact strength observed in polyamide 6 shapes indicates a more ductile nature, rendering the polymer more susceptible to permanent deformation without the occurrence of cracks. In contrast, the composite material demonstrates increased brittleness, resulting in a reduced resistance to dynamic loads.

**Figure 5:**
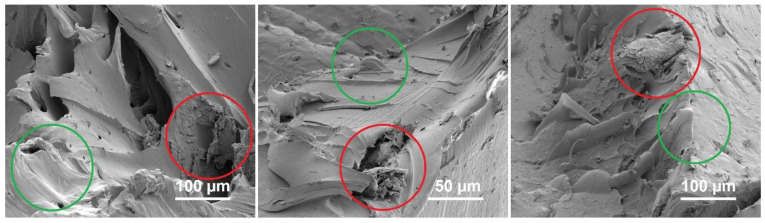
SEM images of the regions where fractures occurred due to mechanical tests in the PA6 + 1 wt-% graphene composite.

In regions where fractures occurred due to mechanical tests on PA6 composites containing graphene, SEM images were captured to demonstrate the impact of graphene flake addition on the rupture behavior of the plates (Figure 5). The figure illustrates the areas where mechanical damage is clearly linked to the presence of the graphene flakes (highlighted with red circles in Figure 5). The disruption of the composite structure in these regions exhibits delamination characteristics, potentially stemming from a weak bond between the flake surface and the polymer. This underscores the necessity for additional functionalization of the flake surface to enhance polymer adhesion. Simultaneously, it can be inferred that the larger size of the lateral flakes contributes to increased mechanical strength (Young’s modulus) during the initial stretching phase. However, under higher forces, it serves as a source of defects and initiates cracking in the composite. The green circles highlight areas in the Figure 5 where the composite exhibits a smooth surface post-damage, indicating a less abrupt disruption of the composite structure.

### 3.3. Antimicrobial Properties

**Figure 6:**
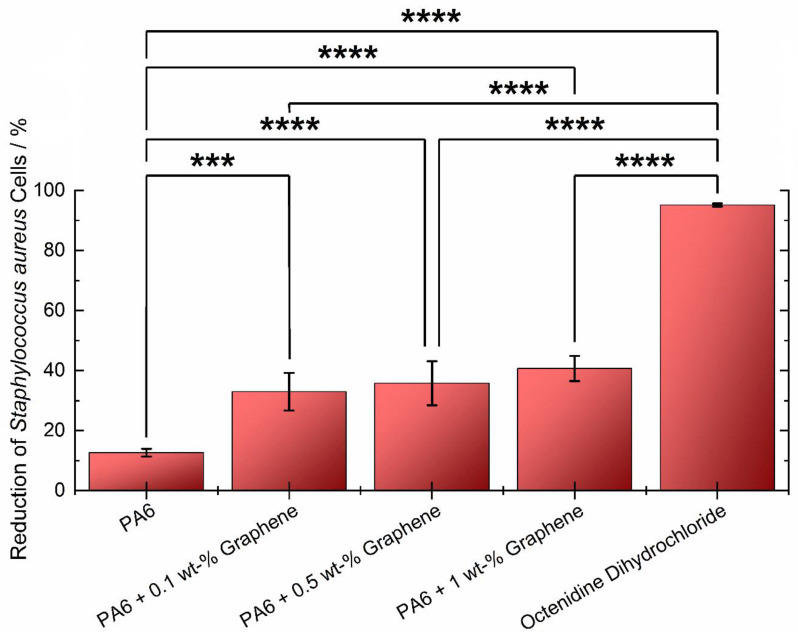
Reduction of *Staphylococcus aureus* cells on the PA6 composites with various concentrations of graphene flakes. Asterisks indicate significant differences between the individual samples.

Taking into account our earlier research, which highlighted the beneficial impact of incorporating graphene into paints on their antibacterial properties [48], we conducted biological tests on the developed composites. This step aimed to further investigate and validate the observed positive effects and delve into the mechanisms underlying the enhanced antibacterial characteristics attributed to the presence of graphene in the composite materials. The tests utilized tetrazolium chloride to monitor the metabolic activities of living *Staphylococcus aureus* cells, inducing a color change. The subsequent dissolution of the resulting red formazan crystals allowed for absorbance measurement, enabling the calculation of bacteria survival rates, and demonstrating the antimicrobial activity of the tested materials. The reference sample of polyamide 6 exhibited the highest absorbance, indicating the presence of a higher amount of red formazan and living bacteria on the surface of the pure polyamide plates. The bacterial reduction for PA6 alone was found to be less than 13% (Figure 6). However, when graphene was incorporated into PA6 samples (0.1 wt%), the reduction surpassed twice that of the reference polymer, reaching almost 33%. Remarkably, polyamide 6 with 1wt% graphene demonstrated the most robust microbial reduction among the tested composites, exceeding 40%, thus showcasing superior bactericidal properties. The mechanism of interaction between the composite and bacteria, leading to bacterial neutralization in this case, may stem from two sources. The first, frequently detailed in the literature, involves the generation of reactive oxygen species (ROS) on the graphene surface. This, upon contact with the bacterial cell membrane, induces damage through what is known as oxidative stress [49]. Another mechanism to consider is mechanical damage, arising from a bacterial cell wall encountering the sharp edges of the graphene flakes protruding from the composite surface [50]. Analyzing the results obtained, it is evident that both mechanisms are highly probable, as evidenced by the enhanced antibacterial activity of the composites with increasing concentrations of graphene flakes within the composite. The introduction of graphene into the polymer effectively inhibited the growth of the *Staphylococcus* strain. These findings underscore the enhanced antibacterial efficacy imparted by the presence of graphene in the polymer matrix. A statistical analysis was performed on the obtained microbiological results, allowing us to determine the influence of the factor (presence of graphene) on the dependent variable (antibacterial activity). Two methods were used for analysis: first ANOVA and then Tukey’s multiple comparison test (*p* < 0.0001). The statistical analysis revealed significant reductions in the number of bacterial cells between the reference and all the graphene samples, all the graphene samples and octenidine, and between the reference and octenidine. Additionally, the analysis showed that the differences in antimicrobial activity between 0.1%, 0.5%, and 1% graphene concentrations are not statistically significant. This suggests that even the lowest concentration of graphene in the composite (applied in this research) exhibits effective antibacterial activity. This finding is significant because reducing the amount of graphene flakes in the polymer decreases the cost of the composite production and demonstrates its potential for advanced antimicrobial applications. Nevertheless, the indication of the lowest concentration of graphene flakes that still maintains the antimicrobial properties within the composite is yet to be elucidated.

## 4. Conclusions

The graphene flakes synthesized through the electrochemical method and integrated into polyamide 6 matrices exhibited promising characteristics for various applications. Characterization techniques confirmed the presence of graphene flakes with desired properties, including interlayer spacing and surface defects, crucial for mechanical reinforcement and antibacterial activity. The SEM analysis revealed a good dispersion of graphene flakes within the composites, minimizing aggregation and enhancing mechanical properties. The mechanical tests demonstrated an increased elastic modulus with graphene incorporation, albeit with a reduction in the impact strength, suggesting a trade-off between stiffness and toughness. The fracture analysis highlighted the role of graphene in enhancing mechanical strength while indicating the need for improved interfacial adhesion. Importantly, the biological tests showcased the significant antibacterial properties of the composites, attributed allegedly to oxidative stress and the mechanical damage induced by the graphene flakes. An improvement in various mechanical properties was observed across all the tested samples, and each composite exhibited significantly enhanced antibacterial properties compared with the pure polymer. The most notable results were obtained with composites containing the highest concentration of graphene flakes (1% by weight). However, balancing the improved mechanical properties with antibacterial activity, alongside considerations of the application suitability and cost (as a higher flake content raises the composite’s price), suggests that the PA6 composite with 0.1 wt-% graphene can effectively be used to produce the antibacterial filament for 3D printing. Overall, these findings underscore the potential of graphene–polymer composites in advanced antimicrobial applications, paving the way for innovative materials with enhanced functionality and performance.

## Figures and Tables

**Table 1:** Mechanical parameters of PA6 + x wt-% graphene composites.

Sample	Average Modulus of Elasticity	Modulus of Elasticity Standard Deviation	Average Tensile Strength	Tensile Strength Standard Deviation	Average Tensile Stress at Failure	Tensile Stress at Failure Standard Deviation	Average Maximum Load	Maximum Load Standard Deviation	Average Deformation at Maximum Load	Deformation at Maximum Load Standard Deviation	Average Strain at Failure	Strain at Failure Standard Deviation
	MPa		MPa		MPa		N		%		%	
PA6	2560	193	69.4	0.7	59.0	3.8	555.3	5.8	5.47	2.9	108.0	10.0
PA6 + 0.1 wt-% graphene	2560	106	66.7	2.1	57.1	7.0	533.6	17.1	3.71	0.2	5.76	0.6
PA6 + 0.5 wt-% graphene	2680	131	72.0	1.6	45.3	1.5	575.8	13.0	3.91	0.2	17.1	1.4
PA6 + 1 wt-% graphene	3020	80	77.5	1.6	50.8	3.5	619.9	13.1	3.82	0.1	17.2	1.2

**Table 2:** Toughness of PA6 + x wt-% graphene composites.

Sample	Average Energy	Energy Standard Deviation	Average Impact Toughness	Impact Toughness Standard Deviation
	J		$\frac{kJ}{m^{2}}$	
PA6	0.277	0.019	8.661	0.592
PA6 + 0.1 wt-% graphene	0.141	0.006	4.411	0.186
PA6 + 0.5 wt-% graphene	0.178	0.020	5.565	0.622
PA6 + 1 wt-% graphene	0.128	0.019	3.983	0.584

## Data Availability

The data presented in this study are available on request from the corresponding author. The data are not publicly available due to privacy.
